# Household Hints and Recipes

**Published:** 1875-04

**Authors:** 


### Household Hints & Recipes.

CHILBLAINS.—A solution of sal-ammoniac in water will often give relief in troublesome chilblains.

---

WATER PROOF CLOTH.—According to an English photographer common white cotton cloth becomes water-proof by being dipped for an instant in dilute sulphuric acid.

---

RESTORNG IVORY.—Discolored ivory may be restored to its original whiteness by cleaning it with a paste composed simply of burnt pumice-stone and water. After cleansing, place the article under a glass in the sun's rays.

---

POISONOUS CHEWING GUM.—A specimen of what is called gum elastic chewing gum has been examined by Prof. Wayne, and the red color of the same was found by him to be vermilion (sulphuret of mercury).

---

FOR CHILBLAINS.—A correspondent of the London *Lancet* recommends a solution of sulphate of copper (4 grains to the ounce) as an application for chilblains. He has found that to succeed when everything else has failed to effect a cure.

---

IMITATION OF TORTOISE-SHELL.—The appearance of tortoise-shell may be given to horn by brushing it over with a paste made of two parts of lime, one part litharge, and a little soap lye, which is allowed to dry. This acts by forming sulphide of lead with the sulphur contained in the albumen of the horn, producing dark spots, which contrast with the brighter color of the horn.

---

TO CLEAN WOODWORK.—Save the tea leaves for a few days, then steep them in a tin pail or pan for half an hour, strain them through a sieve and use the tea to wash all varnished paint. It requires very little "elbow polish," as the tea acts as a strong detergent, cleansing the paint from all impurities, and making the varnish equal to new. It cleans window sashes and oil-cloths; indeed, any varnished surface is improved by its application. It washes window panes and mirrors much better than water, and is excellent for cleaning black walnut picture and looking-glass frames. It will not do to wash unvarnished paint with it. Whiting is unequalled for cleaning white paint. Take a small quantity on a damp flannel, rub lightly over the surface, and you will be surprised at its effects.

GLUE TO RESIST FIRE.—The London *Furniture Gazette* gives this recipe: Mix a handful of quick-lime in 4 oz. of linseed-oil; boil to a good thickness, then spread on plates in the shade and it will become exceedingly hard, but may be easily dissolved over the fire, and used as ordinary glue. It resists fire after being used for glueing substances together.

---

VIENNESE MEERSCHAUM.—The product known as Viennese meerschaum is prepared by mixing 100 parts silicate of soda with 60 parts of carbonate of magnesia and 80 parts of the native meerschaum or pure alumina. This mixture is then pulverized with the greatest care, and passed through a sieve of very fine silk or horse-hair; add water, and boil it for ten minutes; then pour the whole into moulds, placed so that the water may separate easily.

---

HOME REPAIRS OF PLASTERED WALLS.—Small holes in white plastered walls can be easily repaired without sending for the mason. Equal parts of plaster of Paris and white sand—such as is used in most families for scouring purposes—mixed with water to a paste, applied immediately, and smoothed with a knife or a flat piece of wood, will make the broken place as good as new. The mixture hardens very quickly, so it is best to prepare but a small quantity at a time.

---

TRANSPARENT POMADE.—The following is a French recipe:—

Spermaceti, 2 oz.

Castor oil, 5 oz.

Alcohol, 5 oz.

Oil of Bergamot, ½ drm.

Oil of Portugal, ½ drm.

Melt together the spermaceti and castor oil, pour in the alcohol gradually, stop the heat, and add the perfume. Stir well, and pour into glass jars.

---

LIQUID SLATING.—The *Druggist's Circular* recommends the following:—

First, take three parts of the finest ground pumice-stone, and one part of lamp-black; add a little alcohol and grind thoroughly until the mixture appears of a uniform black color. Then dissolve one part of shellac in four parts of alcohol, and stir in the pumice-stone and lamp-black. This liquid makes a surface that can be used with either slate pencils or chalk. It should be kept well stoppered when not in use. If too thick add a little alcohol. Stir well before using. It will be ready for use half an hour after application.

GERMAN APPLE PUDDING.—Butter a pie-dish, and lay in it a layer of bread crumbs, then a layer of good cooking apples pared and quartered, then a layer of good brown sugar, then a very thin layer of finely-chopped suet or little bits of butter, then a layer of bread crumbs, and so on until the dish is filled, taking care to have crumbs at the top. Bake the pudding in a moderate oven for three-quarters of an hour. Before serving, sift sugar on the top.

POTATOES A LA DUCHESSE.—The following is the recipe of a good cook in a private family in Paris: Take five middle-sized, cold, boiled potatoes, grate and mix them with five desert-spoonfuls of flour and a half-penny worth of milk, adding to the mixture two eggs well beaten up; prepare a panful of boiling fat, and drop spoonfuls of the paste into the fat, taking them out the instant they have acquired a delicate golden brown color. With moderate care potatoes thus cooked are delicious.

MOTH PREVENTIVE.—The following for recipe keeping moths out of clothing is a favorite in some families: Mix half a pint of alcohol, the same quantity of spirits of turpentine, and two ounces of camphor. Keep in a stone bottle, and shake before using. The clothes or furs are to be wrapped in linen, and crumpled-up pieces of blotting paper dipped in the liquid are to be placed in the box with them, so that it smells strong. This requires renewing about once a year.

TO COOK APPLES.—1. Stew six or eight good baking apples (pared and cored) until they are tender, let them cool, and mix them with the yolks of two eggs and enough sugar to sweeten them. Spread this mixture on a dish, cover the top with fine bread-crumbs and a small quantity of dissolved butter, and bake for a quarter of an hour. 2. Boil 1½ lbs. of loaf sugar in a pint of water for a few minutes, add 2 lbs. of good cooking apples; let these all boil together until the mixture is tolerably stiff; just before removing it from the fire, add the grated rind of two lemons; press it into moulds which have been previously dipped into cold water (and not wiped); when this *gateau* (as it is called) is turned out on a dish, ornament it with blanched almonds; pour a custard or some whipped cream round it. 3. A very nice way of using apples is to slice some into a dish as for a pie, with some sugar and lemon peel. Make a thick custard of corn flour, milk and sugar, flavored with almonds; pour the custard on the apples, and bake an hour, or till the custard is brown.

TO TAKE OUT BRUISES IN FURNITURE.—Wet the place well with warm water, then take some brown paper five or six times doubled and well soaked in water, lay it on the place, apply on that a hot flat-iron till the moisture is evaporated, and if the bruise is not gone, repeat the process. You will find after two or three applications that the dent or bruise is raised level with the surface. If the bruise is small, soak it well with warm water, and hold a red-hot poker very near the surface, which is to be kept continually wetted, and you will soon find the indentation vanished.

TO GIVE IRON WIRE A SILVERY LOOK.—According to a German recipe, the iron wire is first placed in hydrochloric acid, in which is suspended a piece of zinc. It is afterwards placed in contact with a strip of zinc, in a bath of two parts of tartaric acid dissolved in 100 parts of water, to which is added three parts of tin salt and three parts of soda. The wire remains about two hours in the bath, and is then made bright by polishing or drawing through a drawing iron. The process can also be used for whitening other forms of iron.

CHAPPED HANDS.—The easiest and simplest remedy is found in every storeroom. Take common starch and grind it with a knife until it is reduced to the smoothest powder. Take a clean box and fill it with starch thus prepared, so as to have it continually at hand for use. Every time hands are taken from the suds or dishwater, wipe them, and, while they are yet damp, rub a portion of starch thoroughly over them, covering the whole surface. The effect is magical. The rough smarting skin is cooled and soothed and healed, bringing and insuring the greatest degree of comfort and freedom, from this by no means insignificant trial.

A SIMPLE PLAN OF VENTILATION.—The following simple method for ventilating ordinary sleeping and dwelling rooms is recommended by Mr. Hinton in his "Physiology for Practical Use:"—"A piece of wood three inches high, and exactly as long as the breadth of the window, is to be prepared. Let the sash be now raised, the slip of wood placed on the sill, and the sash drawn closely upon it. If the slip has been well fitted there will be no draft in consequence of this displacement of the sash at its lower part; but the top of the lower sash will overlap the bottom of the upper one, and between the two bars perpendicular currents of air, not felt as draft, will enter and leave the room."

TO COAT IRON WITH BRASS.—The *Scientific Press* describes a method of covering iron wire with brass, without the use of a battery. The process is a very simple one, and consists first in placing the clean, bright wire in a solution of sulphate of copper, when it immediately becomes covered with a thin film of copper. It is then covered with a paste of pure oxide of tin, and heated to a temperature high enough to fuse the copper. Care must, or course, be taken to prevent volatizing the tin.

---

CLEANING BRASS.—If very much oxidized or covered with green rust, first wash it with strong soda and water. If not so very bad, this first process may be dispensed with. Then apply a mixture of one part of common sulphuric acid, and twelve parts of water, mixed in an earthern vessel; wash well, first with clear water, and then with water containing some ammonia, afterwards scouring well with oil and rotten-stone, and using a piece of soft leather and a little dry rotten-stone to give a brilliant polish. In subsequent cleaning, oil and rotten-stone will be found sufficient.

---

RED MARKING-INK FOR CLOTHING.—A red ink for marking clothes, which is not attacked by soap, alkalies, or acids, is prepared by Welger as follows: Enough finely pulverized cinnabar to form a moderately thick liquid is very intimately mixed with egg-albumen, previously diluted with an equal bulk of water, and beaten to a froth, and filtered through fine linen. Marks formed on cloth with this liquid by means of a quill are fixed after they have become dry by pressing the cloth on the other side with a hot iron. The ink will keep in well closed bottles for a long time without separation of the suspended cinnabar.

---

STAINS FOR WOOD.—The following are given in the London *Chemist and Druggist*: *A Green Stain.*—Take 3 pints strong vinegar, 4 ounces best verdigris, ground fine, ½ ounce sap green; thoroughly mix these ingredients. *A Purple Stain.*—Take 1 pound of chipped logwood, 3 quarts water, 4 ounces pearlash, and 1 ounce powdered indigo; boil the logwood in the water for half an hour, then add the pearlash and indigo. *A Cherry Stain.*—Take 3 quarts rain water, 4 ounces annatto; boil in copper kettle till the annatto is dissolved, then put in a piece of potash of the size of a walnut; keep the mixture over the fire half an hour longer, and then it may be bottled for use. *A Mahogany Stain.*—Wash the wood with diluted nitric acid (ten parts of water to one part of nitric acid); for rosewood, glaze the same with carmine or Munich lake. Asphaltum, thinned with turpentine, forms an excellent mahogany color. *A Blue Stain.*—Dissolve copper filings in aqua-fortis; brush the wood with it, and then go over the work with a hot solution of pearlash (2 ounces to 1 pint of water), till it assumes a perfectly blue color.

---

TO CEMENT METAL ON GLASS.—The following cement, recommended by Franke, for fastening prize medals, etc., on the glass of show-cases in the Vienna Exhibition, may be found generally useful in fastening metal on glass securely and rapidly. To an intimate mixture of two parts of finely powdered silver litharge add one part dry white lead, add as much of a mixture of three parts boiled linseed oil and one of copal varnish as will form a doughy mass. It is only necessary to cover the face of the medal with this cement, press it upon the glass, and remove the excess of cement.

---

WRITER'S CRAMP.—P. W. Sheafer, Esq., of Pottsville, Pa., writes as follows to the *Druggists' Circular*: "Noticing the article on 'Writer's Cramp,' in your excellent journal for July, I beg to say, since I abandoned the use of *metalic* pen-holders and adopted those of *gutta-percha* I am no longer troubled with cramp. Formerly the galvanic effect of the metal, as I believe, extended from the fingers to the shoulder, almost disabling me from use of the pen. Some four years since I began the use of gutta percha holders, and now write with ease, though never well. Hoping my experience may benefit others similarly afflicted, I send you this."

---

REMEDIES FOR STINGS.—Among the various cures recommended for bee stings are liquor potassæ, olive oil, vitriol, laudanum, vinegar, honey, saleratus and water, salt and water, soft soap and salt, raw onion, tobacco juice, a paste of clay or flour, the expressed juice of any green leaf, or of the ripe berries of the coral honeysuckle. The poison of a bee being acid, an alkali, must be employed to neutralize it. If, therefore, we were selecting for trial any of the above so-called remedies, we should choose either soft soap or ammonia. But if the individual stung is not very nervous, cold water applied to the wound will be quite sufficient, and it should not be rubbed. One great essential is, if heated, to get cooled as soon as possible, and to avoid becoming heated again for at least two days. Nothing is so apt to make the poison active as heat, and nothing favors its activity less than cold. Let the body be kept cool and at rest, and the activity of the poison will be reduced to a minimum. Any active exertion, whereby the circulation is quickened, will increase both pain and swelling.

---

STRYCHNIA ANTIDOTE.—Olive oil, if administered properly, is said to be an antidote for strychnia.

---

CEMENT FOR LABELS.—A good cement for attaching labels to metal surfaces is composed of ten parts tragacanth mucilage, ten parts honey, and one part flour.

---

SOLDERING BROKEN FILES.—A writer in the *English Mechanic*, who had broken the only half-round file he had by him, says: "After trying to use the broken end (it was broken about the middle) I was about to give up in despair, when I thought I would try soldering it: and, to my surprise, it not only stood while I completed the small job I was doing but is in use still, and will stand all the force such a file needs to have applied to it in ordinary use. I used ordinary solder, Baker's soldering fluid, and a Bunsen burner. The temper at the joining is not in the slightest altered. Of course I was careful not to heat it more than requisite to melt the solder."

---

NOVEL RAT TRAP.—A new manner of catching rats is exciting great interest among householders. A barrel is filled half-full of water. A layer of powdered cork is laid on the surface, and over this a layer of corn meal is sifted. A chair and a box or two are placed unobtrusively in the neighborhood, whereby the rat gains the edge of the barrel. He sees nothing but the meal. He has no innate ideas which teach him to beware of the treacherous foundation on which that tempting surface rests. He sniffs, he leaps, and goes gently down through meal and cork to his watery grave. If any of his friends see him disappear from the edge of the barrel they hasten after him to get their share of the probable plunder, and are in turn taken in by a hospitable death. The plan seems effective as against the rats, but is calculated to destroy their confidence in human nature.

---

APPLES IN IMITATION OF GINGER.—To three pounds of very hard apples take two pounds of loaf sugar, and a quarter of a pound of the best white ginger. Put these in layers (having first sliced the apples in eight pieces and cored them) alternately in a wide-mouthed jar. Next day infuse an ounce of white ginger, well bruised, in about a pint of boiling water; let it stand till the next day. Then put in the apples that have been two days in the ginger. Simmer slowly until the apples look clear. Take great care not to break the pieces.

The following is another recipe, which we find in the London *Garden:* For 4 lbs. of apples take 4 lbs. sugar, one quart of water, and two ounces of best essence of ginger. First pare the fruit, cutting out every particle of core; then shape it to resemble the small kind of preserved ginger. Boil the sugar and water nearly twenty-five minutes, until it is a nice syrup, then put in the apples; be sure not to stir them much; add the essence of ginger (if 2 oz. be not sufficient, add more). It will take nearly an hour to boil, until it becomes yellow and transparent. There will be some pieces that will not clear; put them by themselves, as they will spoil the looks of the rest. It will require skimming.

---

BOTTLING CIDER.—To have good bottled cider, it is necessary first that care should be taken in its manufacture. Apples picked by hand and perfectly ripe and sound are essential to the best quality. They should lie some time after picking. They should then be sorted, their surface wiped dry, and all the rotten fruit rejected. The cider may then be made in the usual manner, by grinding and presssing. The cider should then be stored in a cool place to mature. After three or four months, it should be racked off carefully, and then fined by adding to each hogshead a pound of isinglass finings. In two weeks from the time that the finings are added it should be again racked off, and if found sufficienuly clear and sparkling it is ready for bottling; if not, it should be again find and allowed to stand two weeks. Before bottling the bung should be left out of the casks for ten or twelve hours to permit the escape of carbonic acid gas. The cider may then be placed in bottles, and the corks loosely placed in. The bottles should then be allowed to stand twenty-four hours. The corks may then be driven in and wired down. If the corks are driven in and wired when the cider is first put into the bottles, there will be great danger of breaking the bottles by the accumulating pressure of the gas. All additions of flavoring materials, are, in my opinion, a damage to cider made from a fine quality of fruit, though they may improve juice of a poor quality. If the directions I have given be strictly followed, a delicious cider will be produced.—*American Artisan.*

